# Automatic Neural Processing of Disorder-Related Stimuli in Social Anxiety Disorder: Faces and More

**DOI:** 10.3389/fpsyg.2013.00282

**Published:** 2013-05-24

**Authors:** Claudia Schulz, Martin Mothes-Lasch, Thomas Straube

**Affiliations:** ^1^Institute of Medical Psychology and Systems Neuroscience, University of MuensterMuenster, Germany

**Keywords:** SAD, automaticity, face, fMRI, EEG, emotion

## Abstract

It has been proposed that social anxiety disorder (SAD) is associated with automatic information processing biases resulting in hypersensitivity to signals of social threat such as negative facial expressions. However, the nature and extent of automatic processes in SAD on the behavioral and neural level is not entirely clear yet. The present review summarizes neuroscientific findings on automatic processing of facial threat but also other disorder-related stimuli such as emotional prosody or negative words in SAD. We review initial evidence for automatic activation of the amygdala, insula, and sensory cortices as well as for automatic early electrophysiological components. However, findings vary depending on tasks, stimuli, and neuroscientific methods. Only few studies set out to examine automatic neural processes directly and systematic attempts are as yet lacking. We suggest that future studies should: (1) use different stimulus modalities, (2) examine different emotional expressions, (3) compare findings in SAD with other anxiety disorders, (4) use more sophisticated experimental designs to investigate features of automaticity systematically, and (5) combine different neuroscientific methods (such as functional neuroimaging and electrophysiology). Finally, the understanding of neural automatic processes could also provide hints for therapeutic approaches.

## Introduction

Social phobia, referred to as social anxiety disorder (SAD) in the following, is one of the most frequent psychiatric disorders with a lifetime prevalence of up to 12.1% (Kessler et al., 2005). It is characterized by a “persistent fear of one or more social or performance situations in which the person is exposed to unfamiliar people or to possible scrutiny by others,” accompanied by the fear of embarrassment as the main feature. This leads to avoidance of, or intense anxiety or distress in these situations (Diagnostic and Statistical Manual of Mental Disorders, DSM-IV; American Psychiatric Association, 1994). According to several models and experimental findings, the etiology and maintenance of SAD might be due to automatic cognitive biases in the processing of social information (Beck et al., 1985; Clark and Wells, 1995; Bögels and Mansell, 2004; Morrison and Heimberg, 2013), such as increased automatic processing of angry faces (Gilboa-Schechtman et al., 1999; Mogg et al., 2004). Biased information processing toward clear or ambiguous signals of negative evaluation by others has been repeatedly shown across studies of attention, memory, and interpretation (Heinrichs and Hofmann, 2001; Bögels and Mansell, 2004; Morrison and Heimberg, 2013). Experimental results seem to be best explained by initial hypersensitivity to, followed by subsequent avoidance of the threat stimulus, as proposed in several cognitive hypervigilance-avoidance models (Williams et al., 1988; Amir et al., 1998; Mogg and Bradley, 1998; Bögels and Mansell, 2004; Vassilopoulos, 2005) and models proposing abnormal self-focused attention in social anxiety (Clark and Wells, 1995; Bögels and Mansell, 2004). However, results across studies in SAD are not that consistent, which may depend on the selection of experimental tasks (e.g., emotional stroop, dot-probe, visual search, cognitive load tasks), dependent variables (e.g., reaction times, eye movements, explicit stimuli ratings, autonomic responses, brain responses), processing steps (e.g., early vs. late responses), and stimuli (e.g., different facial expressions, disorder-related words, emotional prosody) (Straube, 2009), as will be described in the following paragraphs.

### Automatic processes: Operationalization and behavioral findings in SAD

Threat processing, not only in SAD but in anxiety disorders in general, is thought to occur involuntarily, non-strategically, and fast, and therefore automatically (Teachman et al., 2012). Automaticity of a process can be defined by several features which may differ independently and may be expressed to various extents in different tasks (Bargh, 1994; Moors and De Houwer, 2006). Bargh (1994) postulates the “four horsemen of automaticity” – awareness, efficiency, intention, and control – as optional and independent features of a process. Awareness or consciousness deals with the phenomenal aspect (i.e., subjective experience) of a process. Efficiency is often defined as an inverse function of the amount of processing resources required to master a task. Intention refers to the controlled initiation of a process, and control describes the ability to avoid, stop, or alter a process once it has been started. However, Moors and De Houwer (2006) argue that there is a strong overlap of intention and control and thus refer to these processes as goal-related features (among others such as goal-dependent, autonomous, and purely stimulus-driven). Taken together, the automatic nature of a process is given when participants do not have particular goals, do not invest a substantial amount of cognitive resources, or do not have awareness in relation to the instigating stimulus, of the process itself, or of its outcome.

Paradigms to investigate unconscious processing of (emotional) stimuli are, for example, backward-masking, binocular rivalry, continuous flash suppression, or the attentional blink paradigm (e.g., Kim and Blake, 2005; Palermo and Rhodes, 2007). The efficiency of the processing of task-irrelevant (emotional) stimuli can be investigated by the attentional load imposed by a distracting main task (Lavie, 1995; Pessoa, 2005). Spatial and feature-based attentional tasks are typical designs to assess goal-related features of automaticity. For example, when the emotionality of an angry face is processed (measured by behavioral performance, autonomic, or brain responses) in a gender discrimination task, this processing occurs at least partially automatically (i.e., uncontrolled and unintentional) as emotion was not task-relevant.

So-called attentional biases, which indicate automatic processing of threat stimuli, can be investigated by paradigms such as dot-probe, modified stroop tasks, or visual search tasks (Bar-Haim et al., 2007; Teachman et al., 2012). Individuals with SAD, but also participants with high scores in trait social anxiety, show biased performance in response to social threat during those tasks (Lundh and Öst, 1996; Gilboa-Schechtman et al., 1999; Mogg and Bradley, 2002; Mogg et al., 2004; Pishyar et al., 2004; Rinck and Becker, 2005) and changed patterns of eye movements when confronted with facial expressions (Mansell et al., 1999; Horley et al., 2004; Garner et al., 2006), even though results vary between studies. For example, findings from dot-probe tasks can be seen as markers of unintentional and uncontrolled processing of threat. In those tasks, participants have to indicate the location of a dot that follows a pair of emotional-neutral-stimuli (e.g., two faces or words, one emotional, and the other one neutral). When responses to the dot are faster at the location indicated by the emotional compared to the neutral stimulus, this is taken as a measure of attentional bias. Dot-probe results in SAD are rather inconsistent (Teachman et al., 2012). Interestingly, *timing* seems to play a crucial role. A vigilance bias for angry compared to happy and neutral faces was found for example only at a presentation duration of 500 ms, but not at 1,250 ms (Mogg et al., 2004). The authors suggest that, with the longer presentation duration, initial orienting might have become overshadowed by avoidance tendencies. Further differences might depend on the use of faces vs. words. Pishyar et al. (2004), for example, found an attentional bias for negative faces but not for negative words in SAD. Findings of Amir et al. (2003) suggest that SAD might be associated with a problem to disengage from threat words. Vassilopoulos (2005) reported that processing of threat words in SAD, as assessed with the dot-probe task, follows a hypervigilant-avoidance pattern. Moreover, Musa et al. (2003) reported an attentional bias to words with the dot-probe task in SAD, when controlling for comorbid depression, suggesting the need for careful further research. Whether attentional biases can occur efficiently, i.e., regardless of cognitive resources (as suggested, for example, in Teachman et al., 2012), has not been tested, as processing resources have not been manipulated. Furthermore, results from subliminal paradigms do not consistently argue for unconscious attentional biases (Mogg et al., 2004; Van Peer et al., 2010).

### Neural correlates of automatic threat-processing in SAD

As described above, behavioral data do not always support the hypothesis of differential automatic processing of threat-related stimuli in SAD compared to healthy controls. This might be due to the fact that behavioral responses as a measure are only partially able to reveal the underlying complex information processing cascade. Increased vigilance, altered perception, (automatic) avoidance tendencies, response selection and execution processes, or coping behavior are often intermingled (Cloitre et al., 1992; Amir et al., 1998; Straube and Miltner, 2006; Straube, 2009). Neuroscientific research might more directly provide information on automatic threat processing in SAD. Along these lines, the investigation of neural activation during exposure to (disorder-related) threat stimuli might not only reveal the neural basis of (reliable) cognitive biases in SAD, but yields the opportunity to investigate hypersensitivity to specific stimuli in SAD even in the absence of distorted overt behavior. From the latter perspective, measures of brain activation can be regarded as an additional method to complement conventional paradigms in experimental psychology, ultimately aiming to answer the question whether and, if so, under what conditions patients suffering from SAD show abnormal automatic processing of social information. The current state of knowledge concerning automatic brain activation to threat in SAD will be described in Section “Automatic Brain Responses to Threat-Related Stimuli in SAD” of this review.

### Scope of the review

This review aims to describe and discuss neuroscientific findings on automatic processing of threat-related stimuli in SAD either as a correlate of altered behavioral performance or in the absence of abnormal performance data. Most research on disorder-related stimuli processing in SAD focused on faces (Machado-de-Sousa et al., 2010; Staugaard, 2010). Facial information such as expression or gaze may have a special relevance in SAD (Schneier et al., 2011): they provide direct, online feedback in social interactions and are of high evolutionary relevance (Öhman and Mineka, 2001). Although the focus of the review is on face processing, we will also discuss research that used other disorder-related stimuli (emotional prosody from voices, verbal stimuli), as these are as important as facial stimuli in social interactions and subject to altered processing in SAD (Quadflieg et al., 2007; Schmidt et al., 2010). It should be noted that recent reviews on SAD focused on a broad range of tasks and methods, but did not specifically address neural mechanisms of automaticity of threat processing (Freitas-Ferrari et al., 2010).

In this review, we will consider both functional imaging studies (fMRI) as well as electrophysiological studies (EEG). While fMRI yields findings with high spatial resolution, the temporal resolution is too slow to give insights into the rapid neural dynamics. In contrast, electrophysiological measures offer very high temporal resolution, but with high spatial uncertainty (Debener et al., 2006). Although a “social anxiety spectrum” with non-clinical and clinical manifestations has been proposed (Schneier et al., 2002; Miskovic and Schmidt, 2012), we focus on participants that meet the clinical criteria for SAD, but also include studies with subclinical samples if these are beneficial for the scope of this review. While Section “Automatic Brain Responses to Threat-Related Stimuli in SAD” reviews findings in detail (including Table 1), Section “General Discussion and Future Directions” offers an integrative discussion concerning the role of experimental tasks, stimulus modalities, emotional expressions, neuroscientific methods, and SAD specificity of the findings. We hope that especially this section stimulates future research on the neurobiology of SAD and automatic threat processing in SAD in particular.

**Table 1:** **Studies investigating automatic processing by means of facial expressions, voices, and words**.

Study	Group	Task	Main results (anxious vs. controls)
**FACES**			
**Studies investigating only differences**			
*fMRI*			
Explicit			
Phan et al. (2006)	SAD vs. HC	Emotion classification	Higher activation of amygdala (angry, fearful, disgusted and sad vs. happy and neutral)
Yoon et al. (2007)	SAD vs. HC	Emotion classification	Higher activation of amygdala, insula (high vs. low arousing angry, fearful, disgusted, sad, happy)
Birbaumer et al. (1998)	SAD vs. HC	Valence, arousal, and intensity judgment	Higher activation of amygdala (neutral faces)
Amir et al. (2005)	SAD vs. HC	Valence judgment	Higher activation of ACC, insula, IFG, PG (disgust vs. neutral)
Cooney et al. (2006)	SAD vs. HC	Valence judgment	Higher activation of right amygdala, lower activation of left amygdala (neutral faces vs. visual baseline)
Klumpp et al. (2010)	SAD vs. HC	Valence judgment	Higher activation of amygdala, insula, OFC, Midbrain, PG (angry, fearful, disgusted)
Labuschagne et al. (2012)	SAD vs. HC	Valence judgment	Higher activation of MPFC, ACC (sad vs. neutral)
Pujol et al. (2009)	HSA vs. LSA	Emotion matching	Higher social anxiety scores associated with higher activation of amygdala (fearful and happy faces vs. shapes)
Labuschagne et al. (2010)	SAD vs. HC	Emotion matching	Higher activation of amygdala (fearful faces vs. shapes, but not angry or happy faces vs. shapes)
Ball et al. (2012)	HSA vs. LSA	Emotion matching	Higher social anxiety scores associated with higher activation of amygdala, insula, ACC (angry and fearful vs. happy)
Sladky et al. (2012)	SAD vs. HC	Emotion matching	Higher habituation of the amygdala, OFC, and Thalamus (anger, disgust, fear, happiness, sadness, surprise, and calm faces vs. shapes)
Klumpp et al. (2012)	SAD vs. HC	Emotion matching	Higher activation of amygdala, insula, IFG (fearful and angry vs. happy)
Free viewing			
Straube et al. (2005)	SAD vs. HC	Free viewing	Higher activation of FG (angry, happy, and neutral faces vs. visual symbol), amygdala (happy and angry vs. visual symbol), insula (angry vs. visual baseline)
Evans et al. (2008)	SAD vs. HC	Free viewing	Higher activation of amygdala, DLPFC; lower activation of ACC (angry vs. neutral schematic faces)
Goldin et al. (2009)	SAD vs. HC	Free viewing	Higher activation of OFC, ACC, PG (angry faces vs. neutral scenes)
Implicit			
Stein et al. (2002)	SAD vs. HC	Gender classification	Higher activation of amygdala, IFG, and MPFC (angry and contemptuous vs. happy, but not fearful and neutral vs. happy)
Campbell et al. (2007)	SAD vs. HC	Gender classification	Higher activation of amygdala and insula, activation later in SAD than in HC (angry, fearful, and happy)
Blair et al. (2008b)	SAD vs. HC	Gender classification	Higher activation of amygdala, MFG, DLPFC, ACC, STS (fearful vs. neutral but not anger vs. neutral)
Blair et al. (2011)	SAD vs. HC	Gender classification	Higher activation of amygdala, ACC (angry and fearful vs. neutral)
Gentili et al. (2008)	SAD vs. HC	n-Back identity	Higher activation of amygdala, insula, STS; lower activation of FG, DLPFC (angry, fearful, disgust, happy, and neutral vs. visual baseline)
*EEG*			
Explicit			
Kolassa et al. (2009)	SAD vs. HC	Emotion classification	Enhanced P1 (angry, sad, happy, and neutral schematic faces)
Van Peer et al. (2009)	SAD vs. HC	Emotion classification	No differences
Moser et al. (2008)	HSA vs. LSA	Emotion classification	Enhanced P3/LPP (anger and disgust vs. happy and surprised)
Sewell et al. (2008)	HSA vs. LSA	Emotional oddball	P3 amplitude associated with level of social anxiety (deviant angry faces, but not happy faces)
Rossignol et al. (2012a)	HSA vs. LSA	Emotional oddball	Enhanced P1 (deviant angry, fearful, disgusted, and happy vs. neutral)
Free viewing			
Mühlberger et al. (2009)	HSA vs. LSA	Free viewing	Enhanced EPN (angry and fearful vs. happy and neutral)
McTeague et al. (2011)	HSA vs. LSA	Free viewing	Sustained ssVEP enhancement (angry, fearful, happy vs. neutral)
Implicit			
Helfinstein et al. (2008)	HSA vs. LSA	Emotional primed dot probe	Enhanced P1, P2 (angry and neutral faces)
Mueller et al. (2009)	SAD vs. HC	Dot-probe	Enhanced P1 (angry faces)
Rossignol et al. (2012b)	HSA vs. LSA	Visual probe task	Enhanced P1, P2 (angry, fearful, disgust, happy, and neutral faces)
Wieser et al. (2011)	HSA vs. LSA	Change detection	Sustained ssVEP enhancement (angry vs. neutral and happy)
Wieser et al. (2012)	HSA vs. LSA	Change detection	ssVEP Face bias to angry faces
**Studies investigating group × task differences**			
*fMRI*			
Straube et al. (2004)	SAD vs. HC	*Explicit:* emotion classification	Higher activation of insula (angry vs. neutral)
		*Implicit:* picture type classification	Higher activation of amygdala, FG, STS, insula, PG (angry vs. neutral)
*EEG*			
Kolassa et al. (2007)	SAD vs. HC	*Explicit:* emotion classification	Enhanced P1 (angry, happy, and neutral schematic faces)
		*Implicit:* color classification	Enhanced P1 (angry, happy, and neutral schematic faces)
Kolassa and Miltner (2006)	SAD vs. HC	*Explicit:* emotion classification	Enhanced N170 (angry faces)
		*Implicit:* gender classification	No differences
**VOICES**			
**Studies investigating group × task differences**			
*fMRI*			
Quadflieg et al. (2008)	SAD vs. HC	*Explicit:* emotion classification	Higher activation of the OFC (anger vs. neutral)
		*Implicit:* gender classification	Higher activation of the OFC (anger vs. neutral)
**WORDS**			
**Studies investigating group × task differences**			
*fMRI*			
Schmidt et al. (2010)	SAD vs. HC	*Explicit:* relevance for social interaction	No differences
		*Implicit:* grammatical type	Higher activation of left amygdala and right OFC

*IFG, inferior frontal gyrus; PG, parahippocampal gyrus; FG, fusiform gyrus; STS, superior temporal sulcus; ACC, anterior cingulate cortex; OFC, orbitofrontal cortex; MPFC, medial prefrontal cortex; DLPFC, dorsolateral prefrontal cortex; SAD, social anxiety disorder; HC, healthy control; EPN, early posterior negativity; LPP, late positive potential; ssVEP, steady state visually evoked potential*.

## Automatic Brain Responses to Threat-Related Stimuli in SAD

Recent meta-analyses and reviews based on functional imaging studies propose a network of regions as the neural underpinnings of different anxiety disorders, including SAD (Etkin and Wager, 2007; Shin and Liberzon, 2010). Different regions seem to have different functions in the various components of fear processing such as the perception of threat, the subjective awareness of fear and anxiety, or the learning, execution, and modulation of fear responses (Straube and Miltner, 2006; Straube et al., 2006; Quadflieg et al., 2008; Shin and Liberzon, 2010; Lipka et al., 2011). Furthermore, activation in different areas seems to follow different time courses and is differentially susceptible to task manipulations modulating efficiency, goal-relatedness, or consciousness (Straube et al., 2011a). In general, both symptom provocation studies in SAD such as public speech (Tillfors et al., 2001) or anticipation of public speech (Lorberbaum et al., 2004) and studies on processing disorder-related stimuli (see below) seem to activate the amygdala. Other regions that have been shown to be activated in SAD comprise the insula (Straube et al., 2004), an area that is involved in the representation of bodily states and anxiety in general (Critchley et al., 2004; Paulus and Stein, 2006; Straube et al., 2006; Etkin and Wager, 2007), and areas in prefrontal cortex (PFC) including orbitofrontal cortex (OFC) (Quadflieg et al., 2008; Goldin et al., 2009) and medial prefrontal cortex (MPFC) (Blair et al., 2012).

It has been proposed that anxiety disorders are characterized by initial hyperactivity of the amygdala to threatening stimuli (LeDoux, 2000; Öhman and Mineka, 2001), with subsequent activation of other regions (Straube and Miltner, 2006; Hofmann et al., 2012). The amygdala has been proposed to mediate automatic, bottom-up processing of emotional, and particularly of threatening stimuli (Öhman, 2005). With its interconnections to various cortical regions as well as the brain stem and hypothalamus, the amygdala plays a central role in an alerting response, in the regulation of the autonomic nervous system, but also in the modulation of perceptual and emotional processing of relevant stimuli (LeDoux, 2000; Pessoa and Adolphs, 2010; Tamietto and De Gelder, 2010; Lipka et al., 2011). Furthermore, activation of the amygdala modulates selective perceptual processing via back-projections to sensory cortices (Vuilleumier et al., 2002; Amaral et al., 2003; Freese and Amaral, 2005). In healthy participants, several studies have demonstrated higher amygdala activation to threat even under attentional distraction (Vuilleumier et al., 2001; Anderson et al., 2003), despite restricted resources (Cornwell et al., 2011; Mothes-Lasch et al., 2013), in backward masking at least for the right amygdala (Morris et al., 1998, 1999; Whalen et al., 1998, 2004; Williams et al., 2006), and under suppression in binocular rivalry (Williams et al., 2004b). Nevertheless, other studies showed no differential activation for negative vs. neutral faces under high perceptual load induced by a main task (Pessoa et al., 2002, 2005b; Bishop et al., 2007; Silvert et al., 2007) or during unconscious processing (Pessoa et al., 2006). These findings emphasize the need to carefully control experimental conditions with respect to different features of automaticity such as difficulty of tasks, saliency of stimuli, quality of the masking procedure (Straube et al., 2010), or expectations of the participants.

Several electrophysiological studies, which allow stronger conclusions on temporal aspects of threat processing, have been conducted in SAD; however, findings are only partially consistent as described below. In general, ERP components measured with EEG are assumed to reflect different stages of processing. These seem to comprise an early, automatic stage associated with enhanced early ERPs to threat-related compared to neutral stimuli [such as P1, P2, N2, or early posterior negativity (EPN)], and a later, more controlled and conscious stage of processing that is associated with enhanced responses of late positive components (starting around 300 ms) (Eimer et al., 2003; Holmes et al., 2003; Liddell et al., 2004; Kolassa et al., 2005; Kiss and Eimer, 2008; Straube et al., 2011b; Michalowski et al., 2012). While early potentials are associated with automatic perceptual analysis and vigilance, late potentials such as P3 and LPC reflect the controlled evaluation of stimuli, motivated attentional processes, and response preparation (Schupp et al., 2000; Straube et al., 2011c).

### Processing of emotional faces

Neurobiological models of face processing (Haxby et al., 2000) suggest that after initial perception of facial features in the inferior occipital gyrus, relatively invariant features (such as identity) of a face are processed in the lateral fusiform gyrus (FG), while changeable aspects of the face activate areas in the superior temporal sulcus (STS), implying a critical role of this region for encoding facial expressions. Crucially, both STS and FG are interconnected with limbic and para-limbic areas, such as the insula, the amygdala, and the OFC. Facial expressions have been shown to modulate the activation of the amygdala (Breiter et al., 1996; Winston et al., 2003b; Fitzgerald et al., 2006) as well as of the extrastriate visual cortex in healthy participants, and this occurs in an automatic fashion (Vuilleumier et al., 2001; Winston et al., 2003a). However, the extent of enhanced automatic processing of aversive faces is a matter of debate (Pessoa, 2005; Straube et al., 2011b).

With regard to automatic neural processing in SAD, we will focus on activations of these regions under different tasks, ranging from explicit emotion processing (e.g., emotion classification, valence judgment, emotion matching) to implicit tasks (e.g., gender classification as well as picture type classification; see Table 1). Similar to emotion classification or emotion judgment, the emotion matching task (Hariri et al., 2002) is a strictly explicit task due to the importance of the facial expression for solving this task. Explicit tasks can show differences in brain responses under conscious and intended processing, and can be opposed to results from implicit, potentially automatic processing. Implicit tasks comprise feature-based or spatial attention paradigms in which the emotional content is not task-relevant. Thus, they can provide more direct hints of automatic processes as the task does not demand the processing of the emotional content, so that they could elucidate dimensions of automaticity such as efficiency (in the presence of a primary task) or the absence of intentionality. A special class of tasks are free viewing paradigms with the instruction just to “watch” the faces. These tasks are supposed to foster “natural” stimulus processing, though one cannot determine what participants actually attend to or do, which severely impedes interpretation. Explicit tasks and free viewing paradigms are not particularly helpful to determine automaticity, as they stimulate conscious and intentional emotion processing and do not allow conclusions on efficiency and control. For the sake of a review on automaticity, studies using implicit paradigms are more suited to investigate features of automaticity, especially those few that compare explicit and implicit emotion processing instead of only group differences. The following summary will stick to this partitioning. To our knowledge, there are no neuroscientific studies investigating awareness and efficiency of emotional face processing in SAD explicitly.

#### fMRI studies

##### Explicit tasks

Studies assessing *emotion classification* show higher activation of the amygdala even to neutral faces (Birbaumer et al., 1998) or higher activation of the amygdala to negative (i.e., angry, disgusted, and fearful) vs. happy faces (Phan et al., 2006) in SAD vs. healthy controls. Yoon et al. (2007) conducted a further analysis of the data by Phan et al. (2006) and found higher activation of the amygdala and the insula to high vs. low arousing emotional (i.e., angry, disgusted, fearful, sad, and happy) faces. In line with these studies, studies assessing *valence judgments* show higher activation of the amygdala to negative (angry, fearful, and disgusted) faces (Klumpp et al., 2010), higher activation of the anterior cingulate cortex (ACC) and insula to disgusted faces (Amir et al., 2005), and higher activation of the ACC and MPFC to sad faces (Labuschagne et al., 2012). Furthermore, Cooney et al. (2006) show a dissociation of the amygdala response to neutral faces, with higher activation of the right, but lower activation of the left amygdala to neutral faces for SAD vs. healthy controls. Studies using the *emotion matching task* showed an association between anxiety ratings and amygdala activation to emotional faces after controlling for FG activation (Pujol et al., 2009), and a correlation of anxiety scores with higher activation of the amygdala, insula, and ACC to emotional faces (Ball et al., 2012). Studies showed higher activation of the amygdala to fearful faces (Labuschagne et al., 2010), and stronger activation of the insula to fearful than happy faces (Klumpp et al., 2012). After initial hyperactivation, strong habituation has been shown for anger, disgust, fear, happiness, sadness, surprise, and calmness in the amygdala, orbitofrontal cortex, and thalamus for SAD but not for healthy controls (Sladky et al., 2012). These studies on explicit emotion processing confirm a neuronal sensitivity of the amygdala, but partially also of the insula, ACC, and prefrontal areas, in SAD to faces in general, especially to negative and arousing faces.

##### Free viewing paradigms

Studies revealed enhanced activation in extrastriate visual areas to happy, angry, and neutral photorealistic faces, enhanced amygdala activation only to happy and angry faces, and enhanced insula activation to angry faces in SAD vs. healthy participants (Straube et al., 2005) as well as enhanced activation of the amygdala to angry vs. neutral but not angry vs. happy schematic faces (Evans et al., 2008). The latter study found higher activation for happy vs. neutral faces only in the FG and insula. These findings demonstrate some inconsistency in the processing of facial expressions in the amygdala and insula under non-restricted experimental conditions.

##### Implicit tasks

A paradigm that ensured attention allocation to another facial information (gender instead of emotion) by incorporating *gender classification* revealed higher amygdala activation to angry and contemptuous compared to happy facial expressions in SAD (Stein et al., 2002), but not for fearful or neutral vs. happy faces. This finding can be regarded as a first hint of automatic, unintentional, and probably efficient processing of negative facial expressions in the amygdala in SAD. A further analysis of the data of Stein and colleagues (Campbell et al., 2007) suggested differential temporal dynamics in individuals with SAD and healthy controls in the processing of fearful, happy, and angry faces, with amygdala and insula activation occurring later in SAD, in the absence of such a timing difference in FG. Delayed amygdala activation to emotional faces in SAD seems counterintuitive. The authors propose this to be a correlate of an atypical orienting response, probably caused by a stronger self-focus and less efficient attention allocation and scanning of environmental information. Thus, this singular finding should be further explored. Furthermore, while one study with angry, fearful, and neutral expressions only found increased activation of amygdala and other regions to fearful faces (Blair et al., 2008b), another one found increased amygdala and ACC activation to angry and fearful faces (Blair et al., 2011). In a *one-back task* on face identity, which also ensured attention allocation to face stimuli, Gentili et al. (2008) found a partially contradicting pattern of higher activation in SAD to angry, fearful, disgusted, happy, and neutral faces (compared to those faces scrambled) in the amygdala, insula, and STS, but reduced activation in the left FG and dorsolateral prefrontal cortex (DLPFC), demonstrating mandatory enhanced processing of emotional content at least in regions associated with emotional evaluation and self-relevance, while the conditions for reduced activation in FG and DLPFC have to be further explored.

To sum up, results from these few implicit tasks are quite heterogeneous concerning the emotion specificity and associated activated brain regions. This could indicate that the effects are not threat-related, but rather an amplified response to faces in general. Nevertheless, due to the tasks applied, the observed effects can be regarded at least as unintentional and probably efficient. However, as the task of gender or identity processing might have been quite easy, the extent of efficient emotion processing cannot be sufficiently determined. Moreover, whether implicit processing is also unconscious or uncontrollable has not been tested so far in fMRI studies.

##### Task effects

The direct comparison of *explicit vs. implicit tasks* in the same study offers the opportunity to investigate automaticity directly and thus address relative automaticity within the same participants, under the same experimental conditions, and with the same stimuli. Astonishingly though, to our knowledge there is only one fMRI study which experimentally varied attention allocation while individuals with SAD were presented with angry and neutral facial expressions (Straube et al., 2004). Individuals with SAD and healthy control participants had to attend either to the facial expression (explicit) or to the picture type (photorealistic vs. schematic) irrespective of the facial expression (implicit task). In the absence of behavioral performance differences between the groups, fMRI data for photographic faces yielded the following findings: in the explicit task, angry vs. neutral faces led to higher activation in all regions in both groups, but stronger effects in SAD compared to controls were only found in the insula. In the implicit task, individuals with SAD showed greater responses to angry vs. neutral faces in the amygdala, insula, FG, STS, and parahippocampal gyrus compared to controls. The comparison of the tasks revealed that while controls generally (in all ROIs) showed stronger activation (i.e., for the contrast angry vs. neutral) in the explicit compared to the implicit task, SAD participants exhibited similar activation under both tasks, suggesting that angry facial expressions were processed automatically – also in extrastriate visual areas – even when this was not required, while healthy participants showed no such mandatory threat-processing. This supports the notion of initial hypervigilance in SAD, with automatic hyperresponsivity under the implicit task only. However, it should be noted that the implicit task once again only offers the opportunity to determine the level of unintentional processing and allows some assumptions on efficient processing, while consciousness and control have not been examined.

So far, fMRI studies with explicit, implicit, and free viewing paradigms yielded mixed results concerning the processing of threat-related faces in SAD in regions as the amygdala and insula, but also ACC, FG, STS, and prefrontal regions. The question remains whether enhanced processing is actually driven by the threat-relevance of the stimuli, or rather by faces as social stimuli *per se*. Interestingly, in the only study that modulated the level of processing within the study (Straube et al., 2004), overall differential processing between the groups only emerged in the implicit condition, suggesting that healthy participants can better stick to task demands, while patients with SAD exhibit mandatory threat-processing even under distraction.

#### EEG studies

##### Explicit tasks

Due to low temporal resolution of fMRI studies, a separation between initial hypersensitivity and subsequent control or avoidance is hard to draw, especially as many studies did not report prefrontal activation. Therefore, ERP studies may be better capable of distinguishing between earlier and later processes and the extent to which these are differentially modulated by emotional content as a function of task-relevance. For ERPs, results from studies with *explicit emotion classification* yield mixed results. One study (Kolassa et al., 2009) showed enhanced P1 amplitudes in response to all (angry, sad, happy, and neutral) schematic faces. This is well in line with the idea that the P1 is modulated by selective attention (Hillyard and Münte, 1984) and could thus indicate a general early attentional bias toward incoming stimuli in SAD. Another study found no differences (Van Peer et al., 2009), while a third study demonstrated larger amplitudes of later P3/late positive potential (LPP) in high socially anxious participants to angry and disgusted faces (Moser et al., 2008). The latter was interpreted as a late negative face bias in high socially anxious individuals that was in contrast to an early positivity bias in low socially anxious participants.

Sewell et al. (2008) used *an emotional oddball task* that requires attending emotion more or less explicitly. Within this task, participants were presented with frequent neutral opposed to rare angry and happy faces. In half of the blocks, participants were requested to respond to angry and to ignore happy faces, and vice versa for the other blocks. The authors report a correlation of P3 amplitude to angry but not happy ignored faces with the level of anxiety. Another emotional oddball study (Rossignol et al., 2012a) demonstrated enhanced P1 amplitudes for deviant (i.e., emotional) faces in participants with high (at least “marked social phobia”) social anxiety scores and a correlation between the level of anxiety and P1 amplitude, while N170 and P3b were not influenced by group. This again argues for an early attentional bias in SAD as indexed by P1. Although the participants were not informed that deviants were determined by emotionality, emotion can be regarded as task-relevant.

##### Free viewing paradigms

A *free viewing* ERP study (Mühlberger et al., 2009) demonstrated no group effects in the N170, but enhanced EPN to fearful and angry faces associated with social anxiety, while an emotional modulation of the LPP observed in low anxious participants was not found in high anxious individuals. By means of steady state visual evoked potentials (ssVEP), a measure of sustained visual activation to certain stimulus types, enhanced early visual processing of angry, fearful, and happy faces was shown (McTeague et al., 2011). These results further show an early perceptual bias toward emotional facial expressions under non-restricted experimental conditions.

##### Implicit tasks

ERP studies that investigated *implicit processing* by means of dot-probe (Helfinstein et al., 2008; Mueller et al., 2009) and spatial cuing (Rossignol et al., 2012b) consistently found enhanced P1 amplitudes to at least angry faces. The two studies that tested high and low socially anxious participants instead of patients diagnosed with SAD (Helfinstein et al., 2008; Rossignol et al., 2012b) additionally found enhanced P2 amplitudes, while no such effect was reported for SAD (Mueller et al., 2009), a finding that should be further substantiated. Results from these implicit tasks once again indicate an early attentional bias in SAD as indexed by enhanced P1, which does not necessarily seem to be related to the threat content. Two ssVEP change detection tasks found enhanced cortical facilitation over visual areas (Wieser et al., 2011) and enhanced frequency amplitudes (Wieser et al., 2012) for task-irrelevant angry faces in individuals with higher social anxiety, demonstrating that this measure also provides insights into preferential visual processing of angry faces in anxious participants. Moreover, the difference between the reviewed explicit and implicit fMRI tasks seems smaller than the one between explicit and implicit EEG tasks, as the attentional bias tasks foster a much more indirect emotion processing with shorter presentation durations. This might explain the absence of later effects, but might yield an even better approach to automatic processing. With very brief presentation durations or masking, these paradigms would allow the investigation of unconscious processing.

##### Task effects

So far, there are only two ERP studies in SAD that *directly compared task effects*. While one study did not find differential effects of the task (Kolassa et al., 2007), another study with photorealistic pictures (Kolassa and Miltner, 2006) which had to be categorized either according to emotion or gender indicated larger right-hemispheric N170 amplitudes in response to angry faces on the emotion identification task in SAD. In contrast to the abovementioned studies, there were no differences between patients and healthy controls in P1 and P2 amplitudes to angry faces. The finding on N170 might indicate abnormalities in early visual threat-processing in SAD. The fact that differential processing occurred in the explicit but not in the implicit task is in contrast to the described fMRI study (Straube et al., 2004).

To conclude, the processing of threat-related facial expressions in SAD seems to be characterized by automatic activation of brain regions associated with initial emotion processing and perception, as well as by enhanced early components associated with attention allocation (see Table 1). However, with both measures (fMRI, EEG), results are rather inconsistent, especially with regards to threat-specificity. The effects occur at least unintentionally, probably even efficiently. As it occurs under conditions in which control of the process is possibly prohibited by the task, it might also be uncontrollable in SAD as compared to healthy controls. Concerning the low number of studies that experimentally varied the automaticity of threat processing in SAD with respect to dimensions of automaticity as consciousness and control, but also efficiency, further research is needed to explore differential activation of the amygdala, insula, visual, and prefrontal areas, as well as early and late ERPs.

### Processing of voice stimuli

The neural network underlying the processing of emotional prosody comprises cortical and subcortical areas (Schirmer and Kotz, 2006; Wildgruber et al., 2009; Brück et al., 2011; Witteman et al., 2012). While the superior temporal regions serve as the cortical input region involved in fast analysis of acoustic features (Rauschecker and Tian, 2000; King and Nelken, 2009), frontal regions are associated with emotional evaluation processes (Ethofer et al., 2009; Leitman et al., 2010). The role of the amygdala might be associated with rapid detection and responding to relevant prosodic features (Sander and Scheich, 2001; Bach et al., 2008b) rather than recognition of specific emotional prosody (Scott et al., 1997). As proposed for the visual modality (Amaral et al., 2003), the amygdala may increase the activation of the STS due to back-projections to sensory areas (Yukie, 2002; Sander et al., 2005). Neuroimaging studies in healthy participants detected activation of the OFC when attention was directed to the emotional information of voices, but not when attention was either directed to other information of the voice or to another spatial location (Sander et al., 2005; Quadflieg et al., 2008). In contrast, the auditory cortex and several subcortical areas showed higher activation to emotional vs. neutral voices irrespective of attention allocation to specific features of the voice (Wildgruber et al., 2005; Ethofer et al., 2006; Bach et al., 2008a; Quadflieg et al., 2008) or specific spatial locations (Sander et al., 2005). However, when attention is directed to the visual modality while hearing emotional voices, higher activation of the amygdala and auditory cortices disappears (Mothes-Lasch et al., 2011). Similarly, a demanding visual task simultaneous to hearing emotional voices diminishes higher activation of the auditory cortex (Mothes-Lasch et al., 2012).

Neural processing of prosodic cues in SAD has as yet only been investigated by one study (Quadflieg et al., 2008; see Table 1). This study explored brain activity when emotional information vs. gender information of a voice was categorized. Thus, emotional information was either task-relevant or -irrelevant. The results showed higher activation to emotional vs. neutral voices in the auditory cortex, insula, amygdala, and other subcortical regions regardless of the task and irrespective of whether participants were anxious or non-anxious. In line with other findings (Wildgruber et al., 2005; Ethofer et al., 2009), the left OFC showed only increased activation to emotional vs. neutral voices when the emotional content had to be attended. However, in anxious participants, higher activation of the right OFC to emotional vs. neutral voices was found in both tasks. Thus, the OFC seems to show an increased activation to threatening voices that is related to the disorder. Interestingly, in line with investigations of facial expressions (Stein et al., 2002; Straube et al., 2004, 2005), differential brain activation occurred in the absence of behavioral effects in anxious and non-anxious participants. In contrast to investigations using angry facial expressions, the study of Quadflieg et al. (2008) failed to reveal any activation differences between groups in the insula or the amygdala. Thus, the processing of threatening voices does not seem to be comparable to the processing of faces and might not rely on hypersensitivity of the amygdala. Nevertheless, the results once again argue for automatic brain responses, leading to different results in patients than in healthy controls. However, these conclusions have to be considered as preliminary and more studies are needed with increased sample size and a wider range of used auditory stimuli.

### Processing of word stimuli

As yet, the processing of word stimuli in SAD has been widely disregarded. However, these stimuli could help to answer the question whether enhanced neural activity in the amygdala to faces is merely due to their evolutionary relevance as proposed by Öhman and Mineka (2001), or whether similar responses also occur for words, as these are highly learned stimuli (stimulus-meaning association) used in social interactions, although they do not implicate threat on their own. Disorder-related words are associated with attentional biases (Amir et al., 2003; Musa et al., 2003; Vassilopoulos, 2005) and rated as being more unpleasant and arousing by individuals with SAD as compared to healthy participants (Schmidt et al., 2010).

Processing of negative vs. neutral words elicits increased amygdala activation (Isenberg et al., 1999; Hamann and Mao, 2002; Maddock et al., 2003) in healthy participants. A recent study (Straube et al., 2011c) reported emotional words to generally elicit increased amygdala activation and that negative compared to positive words elicited higher insula activation. Moreover, direct compared to indirect processing led to enhanced activation for emotional compared to neutral words in dorsomedial PFC and ACC, therefore demonstrating some kind of task-dependence in the activation of these areas.

There are only two studies using word stimuli to investigate neural correlates of SAD. One study presented short statements of praise or criticism either referring to the self or to another person (Blair et al., 2008a) and found enhanced activation to negative statements in medial PFC and amygdala in SAD, but only when the statements were self-referential. The other one is, to our knowledge, the only study which has explored the neural correlates of the processing of threat-related words in SAD (Schmidt et al., 2010) under different task demands (see Table 1). In this fMRI study, patients with SAD and healthy controls had to categorize anxiety-related and neutral words either with respect to their reference to social interaction situations (explicit task) or to their grammatical type (implicit task). In the implicit task, patients showed increased activation in the left amygdala and in right OFC, suggesting a particular role of these areas in the processing of threat-related words when these are not task-relevant. There were no group differences on the explicit task. Nevertheless, symptom severity in patients was significantly correlated with activation in the insula. As the insula is assumed to be associated with monitoring of own emotional states (Straube and Miltner, 2011), this seems to be more expressed when the emotional content is explicitly processed. This finding is well in line with a similar experiment applying facial stimuli (Straube et al., 2004). The finding that group differences emerged especially on the implicit task indicates high vigilance in socially anxious individuals to these stimuli, although they are not as evolutionary relevant as faces.

## General Discussion and Future Directions

We reviewed neuroimaging and electrophysiological studies with respect to the existence and extent of automatic processing of emotional faces and other threat-related social stimuli in SAD (for an overview, see Table 1). While many studies were not designed to address automatic threat processing in SAD explicitly, they nevertheless could demonstrate automatic processing, as far as studies used implicit tasks with respect to the threat-relevance of stimuli. However, these studies provide quite heterogeneous results. Increased activation of the amygdala, insula, and other regions has been shown to all faces regardless of emotion (Gentili et al., 2008) in combination with altered temporal response pattern to emotional faces (Campbell et al., 2007), or to various types of negative facial expressions (Stein, 2002; Blair et al., 2008b, 2011). An fMRI study that directly investigated automatic processes in SAD showed that the higher activation of the amygdala, insula, and other regions to angry vs. neutral faces in SAD persists under implicit task conditions (Straube et al., 2004). Similarly, electrophysiological studies revealed early ERP differences between SAD and healthy controls in P1 either to all facial expressions (Rossignol et al., 2012b) or to negative facial expressions (Helfinstein et al., 2008; Mueller et al., 2009), as well as enhanced ssVEPs to negative facial expressions (Wieser et al., 2011, 2012). ERP studies that have directly investigated automatic processes have yielded mixed results, with either heightened P1 to all facial expressions for SAD vs. healthy controls regardless of the task (Kolassa et al., 2007) or only differential effects for SAD under the explicit task (Kolassa and Miltner, 2006). Furthermore, fMRI studies investigating threat-related stimuli beyond faces revealed increased amygdala activation to social threat words (Schmidt et al., 2010), as well as increased OFC activation to angry vs. neutral prosody and social threat vs. neutral words (Quadflieg et al., 2008; Schmidt et al., 2010). In the following, several aspects of these findings and remaining questions are discussed in relation to brain activation, experimental tasks, stimuli modality, emotional content of stimuli, specificity in SAD, and neuroscientific methods.

### Neuroanatomy of automatic threat processing in SAD

Despite several remaining questions, the reviewed data support models suggesting a role of the amygdala for automatic threat processing in SAD. The role of the amygdala might be to potentiate vigilance to relevant stimuli and to process the emotional valence of stimuli (Pessoa and Adolphs, 2010; Lipka et al., 2011; Straube et al., 2011b). It should be noted that different subregions of the amygdala have been proposed to subserve different functions. Attentional functions have been ascribed to the central nucleus within the dorsal amygdala, while responses to stimulus valence seem to mainly activate the ventral amygdala that covers the basolateral parts of the amygdala, which have been shown to be more strongly involved in stimulus processing than in the organization of behavioral responses (Kim et al., 2004; Hoffman et al., 2007; Boll et al., 2011). This dissociation of dorsal and ventral parts within the amygdala is also in line with a recent meta-analysis by Mende-Siedlecki et al. (2013), in which the authors found differences in the response of neuronal populations in ventral and dorsal parts of the amygdala for valence (ventral amygdala) and salience (dorsal amygdala) of a facial expression. Future studies should use high-resolution fMRI to investigate the role and task-dependence of activation of specific subregions of the amygdala in SAD. Furthermore, several fMRI and EEG studies found increased activation in visual areas in response to angry faces in SAD (Straube et al., 2005; Wieser et al., 2011, 2012), especially under implicit conditions (Straube et al., 2004). This supports the hypothesis of increased sensory processing of disorder-related threat. Since the amygdala modulates selective perceptual processing via back-projections to sensory cortices (Vuilleumier et al., 2002; Amaral et al., 2003; Freese and Amaral, 2005), the activation in sensory areas might at least be partially boosted by the amygdala.

Besides the amygdala, several other areas seem to be involved in threat processing in SAD. Medial and lateral prefrontal areas and ACC have been suggested to be involved in cognitive-emotional interactions (Straube and Miltner, 2011). In general, these areas seem to be relevant for higher cognitive appraisal processes, for the control and regulation of behavioral emotional responses, and partially also for the generation of autonomic emotional responses. Especially ventral parts of the PFC and OFC have been implicated in the control of emotional responses (Ochsner and Gross, 2005). These areas seem to be involved in controlled and explicit threat processing. Self-referential attention in SAD might also be an automatic response (Clark and Wells, 1995) and might be associated with MPFC (Blair et al., 2008a).

Several studies found OFC activation to threat in SAD, even during implicit tasks (Quadflieg et al., 2008; Schmidt et al., 2010; see Table 1). The OFC has previously been proposed to evaluate sensory signals to enable appropriate affective and behavioral responses (Damasio et al., 1994; Rolls, 2000). More specifically, its role in the representation of rewards and punishments guiding learning and behavioral adjustments has repeatedly been pointed out (Damasio et al., 1994; Rolls et al., 1994; Rolls, 2000). A lack of orbitofrontal modulation due to brain damage results in severely disturbed social behavior and inappropriate impulsiveness (Damasio et al., 1994; Rolls et al., 1994; Hornak et al., 2003). Thus, in social anxiety, the functional role of orbitofrontal activation might be to support avoidance behavior or to inhibit actual behavior in the case of perceived social threat (Veit et al., 2002; Quadflieg et al., 2008; Schmidt et al., 2010).

Furthermore, several studies found activation of the insula to social stimuli, even during implicit tasks (Straube et al., 2004; Campbell et al., 2007; Gentili et al., 2008; see Table 1). This brain region is strongly involved in interoception and representation of bodily states and might support aversive feelings by the perception of bodily states of arousal (Straube et al., 2004). However, findings are inconsistent and the role of task conditions is unclear. Even if insula responses can be detected during implicit tasks (Straube et al., 2004), activation in this area increases with increasing attention to own emotions (Straube and Miltner, 2011) and is more strongly associated with symptom severity of SAD during explicit emotion tasks (Schmidt et al., 2010).

Taken together, there might be different processing stages associated with different activation profiles and different cognitive-emotional processes, e.g., avoidance and regulatory strategies and inhibition of the amygdala following initial hypervigilance to threat (Hofmann et al., 2012). Future studies should investigate the time course and role of different areas for automatic and non-automatic processes in more detail.

### Role of task conditions

There are only a few systematic attempts to investigate automatic neural processes in SAD by means of task modulation (see Table 1), which allows separating different information processing modes (Straube and Miltner, 2011). While implicit processing modes are related to rather automatic responses to emotional stimuli, explicit processing requires that participant’s attention is directed to the emotional significance of stimuli or to one’s own emotional involvement, i.e., to own feelings (LeDoux, 1996; Damasio, 1999). As described, these different levels of information processing seem to depend partially on different neural systems (LeDoux, 1996; Damasio, 1999; Phan et al., 2002). To elucidate the specific role of a brain region as a function of the information processing mode, it is necessary to systematically vary the level of evaluation of emotional stimuli and to control for other factors (see also Lieberman et al., 2007). For example, in line with a recent study (Straube and Miltner, 2011), future studies could systematically manipulate participants’ attention to stimuli and emotional relevance of stimuli.

Furthermore, the extent of automatic processing in terms of efficiency could be investigated by varying demands of a main task while threat-related stimuli are presented and thus serve as distractors. As proposed by Lavie (1995, 2005), attentional resources available for the processing of distractors should decline with increasing perceptual demands. Thus, distracting stimuli should not be processed under high attentional load. In line with this prediction, higher activation of the amygdala and partly of sensory cortices to threat-related faces diminished under high attentional load in healthy participants (Pessoa et al., 2002; Bishop et al., 2007; Silvert et al., 2007). However, for example spider phobics did not show reduced activation of the amygdala and sensory cortices to disorder-related stimuli even under high attentional load (Straube et al., 2011a). These results suggest efficient processing of threat-related stimuli in anxious individuals. Similar studies should be conducted in SAD.

The impact of awareness could be examined by studies using backward masking (but see for methodological problems Pessoa et al., 2005a, 2006; Straube et al., 2010) or binocular rivalry (Pasley et al., 2004; Williams et al., 2004a). For specific phobia, a link between vigilance to threat and amygdala responses to subliminal threat has been proposed (Lipka et al., 2011). Whether this holds also true for SAD remains to be elucidated.

Only some studies investigated neural correlates of attentional biases (Kolassa et al., 2007; Helfinstein et al., 2008; Mueller et al., 2009; Rossignol et al., 2012a) and found generally enhanced P1 amplitudes in SAD irrespective of the existence of behavioral effects. Therefore, the relation should be further substantiated by correlations or regressions between the behavioral data and neural activation. So far, ERPs have only been investigated from P1 onward. However, very recent MEG results (Steinberg et al., 2012) indicate even earlier automatic differential processing of emotional stimuli in frontal and occipito-temporal regions (50–80 ms). These rapid effects should also be investigated to test the hypothesis of rapid, non-intended threat-processing in SAD.

Finally, processes beyond initial hypervigilance to threat might also be triggered automatically. These include avoidance of external threat or increased self-focused attention. It is likely that these processes are at least partially automatic, probably as a highly learned reaction in response to threat stimuli in SAD. Designs that vary cognitive resources or intentional focus might investigate automaticity of these processes.

### Faces and more

Due to the large number of studies focusing on emotional face processing in SAD, the question arises whether automatic processes are specific for faces. Emotional prosody can also be regarded as a disorder-relevant stimulus, which provides direct hints of approval or disapproval in SAD. In contrast to findings for faces, the study of Quadflieg et al. (2008) failed to reveal differences in emotional voice processing in the amygdala (but found automatic activation of the OFC). Whether these results indicate dissimilarities of processing of threat-related stimuli in the visual and auditory modality, or whether the task was not able to reveal differences, needs to be further explored. Implicit processing of threat-related words in SAD (Schmidt et al., 2010) demonstrated enhanced activation of the amygdala and OFC, suggesting that these stimuli, although not evolutionary relevant, are processed as automatically as faces. Thus, automatic brain processes do not seem to be specific for faces. The dominance of a certain modality in SAD could best be investigated by combined experimental designs that use faces, words, and voices as stimuli. Furthermore, the comparison of emotional stimuli presented in different modalities could reveal insights in preconditions for the activation of supramodal emotion representations. There might also be differences between sensory modalities in the sensitivity to specific emotions. Finally, social interactions obviously do not exclusively rely on faces, voices, and words. Gestures are also powerful signals eliciting similar emotional responses as faces (Magnée et al., 2007; De Gelder, 2009) and neural responses to such cues in SAD might be investigated in future studies.

### Specificity of threat-related stimuli

Whether certain emotions in SAD are more relevant than others is a matter of debate. Remarkably, different studies compared different sets of emotional expressions and reported inconsistent effects for negative, positive, and neutral stimuli (see Table 1). While some studies on SAD showed abnormal processes with regard to angry faces, healthy participants show predominantly higher activation of the amygdala and FG to fearful faces (Whalen et al., 2001). Additionally, most studies on trait and/or state anxiety reveal sensitivity and automatic activation of the amygdala to fearful faces (Etkin et al., 2004; Bishop et al., 2007). While both fearful and angry faces provide information of threat, the source information concerning the threat differs between these stimuli (Whalen, 1998). Confronted with an angry face, the source of the threat is the person showing that expression, and the addressee the person perceiving that face (in the case of direct gaze). In contrast, fearful faces only provide information about the presence of threat without the information of its source, thus providing a more ambiguous or diffuse signal of threat. Amygdala responses might be modulated by the relevance of the stimuli for the specific disorder, e.g., stimuli of rejection for SAD and stimuli of ambiguity for anxiety in general. However, results show higher activation to angry but not to fearful faces (Stein et al., 2002), which is partially in contrast with another study (Blair et al., 2011) that found increased activation to both angry and fearful faces in SAD, and an earlier study (Blair et al., 2008b) that only found increased activation to fearful faces.

Furthermore, some studies have even reported enhanced activation to happy faces in SAD (Straube et al., 2005). This might be due to the use of a free viewing paradigm. Probably, under restricted conditions, only the processing of threat-related stimuli is boosted. Higher amygdala activation even to neutral faces (Birbaumer et al., 1998) might be explained by a negative interpretation bias in SAD for ambiguous signals of threat. Another explanation might be that SAD is characterized by generally modified face processing without a specific focus on threat-relevance. Thus, it remains to be answered whether effects in SAD are disorder-related, corresponding to specific emotions, or whether they are driven by general factors such as ambiguity, valence, or just arousal or intensity. The latter is in line with the finding of higher amygdala activation to high vs. low intensity expressions only in SAD (Yoon et al., 2007). Methodologically, it is difficult to distinguish between valence and arousal, since threat-related faces are more arousing than happy faces (see also Straube et al., 2005). Future studies should carefully compare a broad range of emotional expressions and simultaneously control for valence, arousal, and intensity.

In this paragraph, we discussed threat-specificity in face processing. Nevertheless, the considerations apply also to prosody and word stimuli with respect to the role of different emotions as well as the role of valence, arousal, and intensity. Needless to say, there is also a bulk of other aversive stimuli such as odors that could be investigated.

### Disorder specificity

Future studies should aim to investigate whether neural automatic processing of threat-related social stimuli found in SAD is specific to this disorder, or whether there are common mechanisms in other anxiety disorders or psychopathologies. The processing of threat-related stimuli in different anxiety disorders might differ in the extent of automatic processing. For example, Phan et al. (2006) show a correlation of the activation of the amygdala to negative faces with severity of social anxiety symptoms but not with state or trait anxiety, and Rossignol et al. (2012b) show that heightened P2 and P3 to angry faces is associated with social anxiety but not with trait anxiety, indicating differences in early automatic processing. Other studies suggest a dissociation between SAD and generalized anxiety disorder (Blair et al., 2008b). While patients with SAD showed increased amygdala activation to fearful faces related to their reported level of anxiety, patients with generalized anxiety disorder had decreased activation to these faces, but increased activation to angry faces in a lateral region of the middle frontal gyrus, which was in turn related to their level of reported anxiety. A meta-analysis on SAD, specific phobia, and post-traumatic stress disorder (PTSD; Etkin and Wager, 2007) showed hyperactivation of the amygdala and insula more frequently in SAD and specific phobia than in PTSD, while the latter showed reduced activation in dorsal and rostral ACC and ventromedial prefrontal cortex during negative emotional processing. Even on the behavioral level, it has been suggested that anxiety disorders are associated with uncontrollable, unintentional, and unconscious processing of negative information, whereas major depressive disorder is associated with only uncontrollable processing (Teachman et al., 2012), demonstrating the need for carefully distinguishing between different aspects of automaticity. In each case, correlations between measures of automatic processes and symptom severity would improve interpretations concerning the disorder specificity of the observed effects.

### Changing automatic responses to threat

Some training studies in the style of dot-probe tasks have focused on attention allocation to positive stimuli in anxiety. They found positive post-training effects on self-reported anxiety (Schmidt et al., 2009), behavioral and physiological (skin conductance) measures (Heeren et al., 2012), as well as response times and ERP amplitudes (Eldar and Bar-Haim, 2010). The effects of these studies may rely on stronger control of distracting stimuli. However, the neural basis of these therapy effects is unclear. One could assume changes in amygdala reactivity or PFC involvement. Therefore, it would be interesting to apply the abovementioned tasks in therapy-evaluation studies with respect to the changeability of automatic brain responses and the relationship between them and therapy outcome. The initial extent of automatic brain responses might serve as a predictor for therapy outcome (Doehrmann et al., 2013), and the individual changeability of automatic processes might probably serve as a predictor for longtime effects of therapy.

## Conclusion

We reviewed the current evidence for automatic neural processing of threat-related stimuli in SAD by means of neuroimaging and electrophysiological studies to shed more light on the spatial and temporal basis of these processes. We incorporated studies with different stimuli, modalities, and experimental designs, and reported evidence for automatic activation of the amygdala, insula, and sensory cortices as well as in early electrophysiological components under different tasks and experimental conditions (see Table 1). As systematic studies, especially on the nature and extent of automatic processing, are still widely lacking, we suggested experimental designs aimed at providing a more comprehensive view of this disorder. These should: (1) use different stimulus modalities to explore face specificity, (2) examine different emotional expressions, (3) compare different forms of anxiety, (4) use more sophisticated experimental designs to investigate features of automaticity systematically (including direct associations with attentional biases), and (5) combine neuroscientific methods (such as functional neuroimaging and electrophysiology). These approaches might further increase the understanding of SADs and might also give useful hints for therapeutic approaches. Studies might test whether automatic brain responses are affected by successful psychotherapy or whether such responses predict therapy success in the short or long term.

## Conflict of Interest Statement

The authors declare that the research was conducted in the absence of any commercial or financial relationships that could be construed as a potential conflict of interest.
